# Cancer Cell enters reversible quiescence through Intracellular Acidification to resist Paclitaxel Cytotoxicity

**DOI:** 10.7150/ijms.46034

**Published:** 2020-06-29

**Authors:** Min Jia, Dianpeng Zheng, Xiuyun Wang, Yongjun Zhang, Sansan Chen, Xiangsheng Cai, Lijun Mo, Zhiming Hu, Hongwei Li, Zhongxin Zhou, Jinlong Li

**Affiliations:** 1 Institute of Biotherapy, School of Laboratory Medicine and Biotechnology, Southern Medical University, Guangzhou, Guangdong, China.; 2 Department of Vascular Surgery, The Third Affiliated Hospital, Southern Medical University, Guangzhou, Guangdong, China.; 3 Department of Urology, The First Affiliated Hospital, Guangdong Pharmaceutical University, Guangzhou, Guangdong, China.; 4 Clinical Laboratory, The First Affiliated Hospital, Guangdong Pharmaceutical University, Guangzhou, Guangdong, China.

**Keywords:** chemo-resistance, quiescence, intracellular acidification, NHE1, MCM7

## Abstract

Cancer cells can enter quiescent or dormant state to resist anticancer agents while maintaining the potential of reactivation. However, the molecular mechanism underlying quiescence entry and reactivation remains largely unknown. In this paper, cancer cells eventually entered a reversible quiescent state to resist long-term paclitaxel (PTX) stress. The quiescent cells were characterized with Na^+^/H^+^ exchanger 1 (NHE1) downregulation and showed acidic intracellular pH (pH_i_). Accordingly, decreasing pH_i_ by NHE1 inhibitor could induce cell enter quiescence. Further, acidic pH_i_ could activate the ubiquitin-proteasome system and inhibiting proteasome activity by MG132 prevented cells entering quiescence. In addition, we show that after partial release, the key G1-S transcription factor E2F1 protein level was not recovered, while MCM7 protein returned to normal level in the reactivated cells. More importantly, MCM7 knockdown inhibited G1/S genes transcription and inhibited the reactivated proliferation. Taken together, this study demonstrates a regulatory function of intracellular acidification and subsequent protein ubiquitination on quiescence entry, and reveals a supportive effect of MCM7 on the quiescence-reactivated proliferation.

## Introduction

Quiescent cancer cells are the major obstacle for successful anti-cancer treatment because of their relative insensitivity to almost all current anti-cancer strategies 1. Quiescent cancer cells are frequently observed in patients who have undergone chemo- or radio- therapy in various type of cancer 2-5. Also, the disseminated tumor cells (DTC), which accounts for metastatic lesions, are frequently demonstrated to be quiescent. More importantly, such quiescent cells can be reactivated to growth, leading to local or metastatic recurrence. Considering the super drug resistant capability of quiescent cancer cell, the ultimate goal would be, if not to eliminate quiescent cells, to prevent their reactivation 6.

Homeostasis of pH in mammalian cells plays fundamental roles in regulating essential cell events, such as proliferation, apoptosis, metastasis and differentiation 7. The intracellular pH (pH_i_) is tightly regulated via multiple plasma membrane ion transport proteins with a near-neutral range (7.0-7.2) in most normal cells 8. Deregulation of pH_i_ has been observed in cancers. Na^+^/H^+^ exchanger 1 (NHE1), the major regulator of pH_i_, is frequently upregulated in proliferating cancer cells, resulting in a reversed pH gradient with acidic extracellular pH (pH_e_: 6-6.8) and alkaline pH_i_ (7.3-7.6) 9, 10. All these studies indicate a close relationship between pH_i_ and cell proliferation. However, the characteristics and the potential regulatory role of pH_i_ in quiescent cancer cells are still unclear.

Minichromosome maintenance proteins (MCM2-7) are components of the pre-replicative complex and function as helicases during DNA replication. However, their excessive abundance and distribution patterns in chromatin present the so-called “MCM paradox” 11. Excessive MCMs more than replication origins are loaded onto chromatin and distribute over non-replicated DNA 12. This indicated that MCM have additional function except for DNA helicases. It have been suggested that the excessive chromatin-bound MCM are required for human cells to survive replicative stress by firing backup origins 13, 14. Therefore, it is possible that MCM may play roles in the reactivated proliferation of cancer cells under replicative stress.

In the present study, a reversible quiescent cancer cells were induced by long-term PTX treatment. We studied the relation between intracellular pH and quiescence entry, and characterized the reactivated proliferative profile of the quiescent cancer cells. We reveal a causative effect of acidic pH_i_ on quiescence entry and demonstrate that MCM7 play an important role in the quiescence-reactivated proliferation.

## Materials and Methods

### Reagents, cell culture and drug treatment

PTX, norcantharidin and cisplatin were purchased from Sigma-Aldrich. Vincristine and Cariporide (NHE1 inhibitor) were purchased from Selleck Chemicals. MG132 (proteasome inhibitor) was purchased from MedChemExpress Chemicals. Hepatocellular carcinoma HepG2 and Human bladder cancer UMUC-3 cell lines were routinely maintained in DMEM medium (Gibco BRL, Grand Island, NY, USA). The medium contains 10% fetal bovine serum, penicillin (100 U/ml) and streptomycin (100μg/ml). Cells were cultured at 37 °C in a balanced air humidified incubator with an atmosphere of 5% CO_2_. HepG2 and UMUC-3 cells were continuously treated with 40nM and 80nM PTX respectively for 7 days. The residual cells were then partially released into medium with half of the initial concentration of PTX.

### Cell viability measurement

Cell viability was quantified using Cell Titer 96 Aqueous cell proliferation assay (MTS) (Promega). The results were expressed as mean ± SD viable cells relatively to drug vehicle alone (considered as 100% viability).

### Quantitative real-time PCR

Total RNA was isolated from cells using Trizol® reagent (Invitrogen, USA), and was used to generate cDNA with PrimeScript^™^ RT Master Mix (Takara, China). Real-time PCR was performed using the Applied Biosystems® 7500 Real-Time PCR Systems (Thermo Scientific™, USA). The data were analyzed using 7500 software. Real-time PCR quantification used 2^-ΔΔCt^ method against the β-actin for normalization. Real-time PCR primers were listed in Table S1.

### EdU incorporation assays

Cell proliferation was detected with 5-ethynyl-2′-deoxyuridine (EdU) labeling/detection kit (Ribobio, Guangzhou, China). Cells were planted in 24 well plates (1×10^5^ cells/well). EdU labeling medium (1:1000) was added into wells and incubated for 2 hours as the protocol indicates. After EdU staining, cells were counter stained with Hoechst33342. The percentage of EdU^+^ cells was calculated from five random fields each in three wells.

### Western blotting

Total proteins from cells were extracted with ice-cold lysis buffer (50mM Tris-HCL pH 7.5, 150 mM NaCl, 1% NP40, 1 mM PMSF, and 10 units/ml aprotinin) for 5-10 min, then centrifuged at 12000 rpm form 10min at 4 °C. Proteins (about 20μg) were separated by 12% SDS-PAGE and transferred onto polyvinylidene fluoride (PVDF) membranes and incubated overnight at 4 °C with antibody against Cyclin D1, Cyclin E1, Cyclin B1, ubiquitin (Proteintech, China), RB, E2F1 or GAPDH (Bioworld technology, China). After washing with Tris-buffered saline supplemented with 0.1% Tween 20, the membranes were incubated with HRP-conjugated IgG at room temperature for 1 h. Signal detection was carried out with an ECL system (Millipore, Billerica, MA, USA).

### Analysis of apoptosis and flow cytometry analysis

Quantification of apoptosis was performed with Annexin V and Propidium Iodide (PI) staining according to the manufacturer's (KeyGEN). Briefly, 1×10^5^ cells were resuspended in Annexin V binding buffer and stained with Annexin V-FITC and Propidium Iodide (1 μg/ml). After incubation at room temperature for 15 min, the apoptotic cell was quantified by flow cytometry. For cell cycle analysis, cells were fixed in 70% ethanol overnight at 4 °C and then stained with propidium iodide (PI) (50 μg/ml PI, 100 μg/ml RNase, 0.05% Triton X-100 in PBS) for 30 min. DNA content was analyzed by using flow cytometry. For the detection of CD34^high^ or CD133^high^ population, cells were incubated with monoclonal antibody: CD34-FITC and CD133-FITC (eBiosciences) on ice for 20 min in the dark. At least 1× 10^4^ cells per sample were analyzed with the flow cytometry.

### Intracellular pH Measurement

Measurements of pHi were performed using the pH-sensitive fluorescent dye 2,7-bis- (2-carboxyethyl)-5-(and-6)-carboxyfluorescein (BCECF-AM, Invitrogen) 15. In brief, Cells were incubated at room temperature with Ringer solution (154 mM NaCl, 2.2 mM CaCl_2_, 5.6 mM KCl, 2.4 mM NaHCO_3_, 2 mM Tris-HCl, pH 7.4) containing 2 µM BCECF-AM (Invitrogen) for 10 min. The fluorescence intensity was detected by lympus Provis fluorescence microscope (Nikon Eclipse Ti-SR) in same exposure time and calculated by Image-Pro Plus 6.0 software. pH calibration was performed after each experiment by the nigericin (MedChemExpress) technique. A ten-point in situ pH calibration (6.4 to 8.2) was performed in sodium-free calibration buffer (125mM KCl, 1 mM MgCl_2_, 1 mM CaCl_2_, 20 mM HEPES and 10 µM nigericin).

### Senescence β -Galactosidase Cell Staining

β‐Galactosidase staining was performed according to the manufacturer's instructions as previously described 16 (Cell Signaling Technology, USA). Briefly, the cells were washed with PBS and fixed at room temperature. Each well was filled with 1 ml β‐Galactosidase Staining Solution. The plate was sealed with a parafilm and incubated at 37 °C overnight in a dry incubator (no CO_2_). The percentages of SA-β-Gal-positive cells were identified as bluish green-stained cells under a phase-contrast microscope.

### MCM 7 siRNA

The MCM7-targeting siRNAs (si-MCM7: 5′GAGTTGGTGGACTCAATTT3′) was purchased from Ribobiotech (Guangzhou). For the transfection of siRNA, cells (5×10^5^) were seeded into 6-well plates and then were transfected with siRNA in diluted Lipofectamine containing Opti-MEM Medium (Invitrogen) according to manufacturer's protocol. Non-targeting siRNA was used as control.

### Chromatin Immunoprecipitation (ChIP)

Chromatin Immunoprecipitation assay was performed using Pierce™ Agarose ChIP Kit (Thermo Scientific™) following manufacturer's protocol. The immunoprecipitation of DNA-protein complexes was achieved with antibodies directed against E2F1 (Cell Signaling Technology) or IgG. The abundance of the candidate sequences was measured by quantitative PCR amplification. The primers for the promoter were: CCND1 (Cyclin D1): F: 5'-ATGCGGAATCCGGGGGTAAT-3, R: 5'-AGGGTGCTCACAGCAAGATG-3' 17; CCNE1 (CyclinE1): F: 5'-TTGCAGAGCAGCAGCCAGGG-3', R: 5'-CGGCGCACTGCGTTGAAACC-3' 17. Values reflecting chromatin enrichment are reported as the percent of input.

### Statistical analysis

Data were presented as the mean ± S.D. from at least three separate experiments. Statistical analyses were performed using GraphPad Prism 5 (GraphPad Software, Inc., CA, USA). Multiple group comparisons were performed using ANOVA with a post hoc test for the subsequent individual group comparisons. *p* < 0.05 was considered to be significant.

## Results

### Cancer cells enter a reversible quiescent state under long-term PTX stress

**Figure 1:**
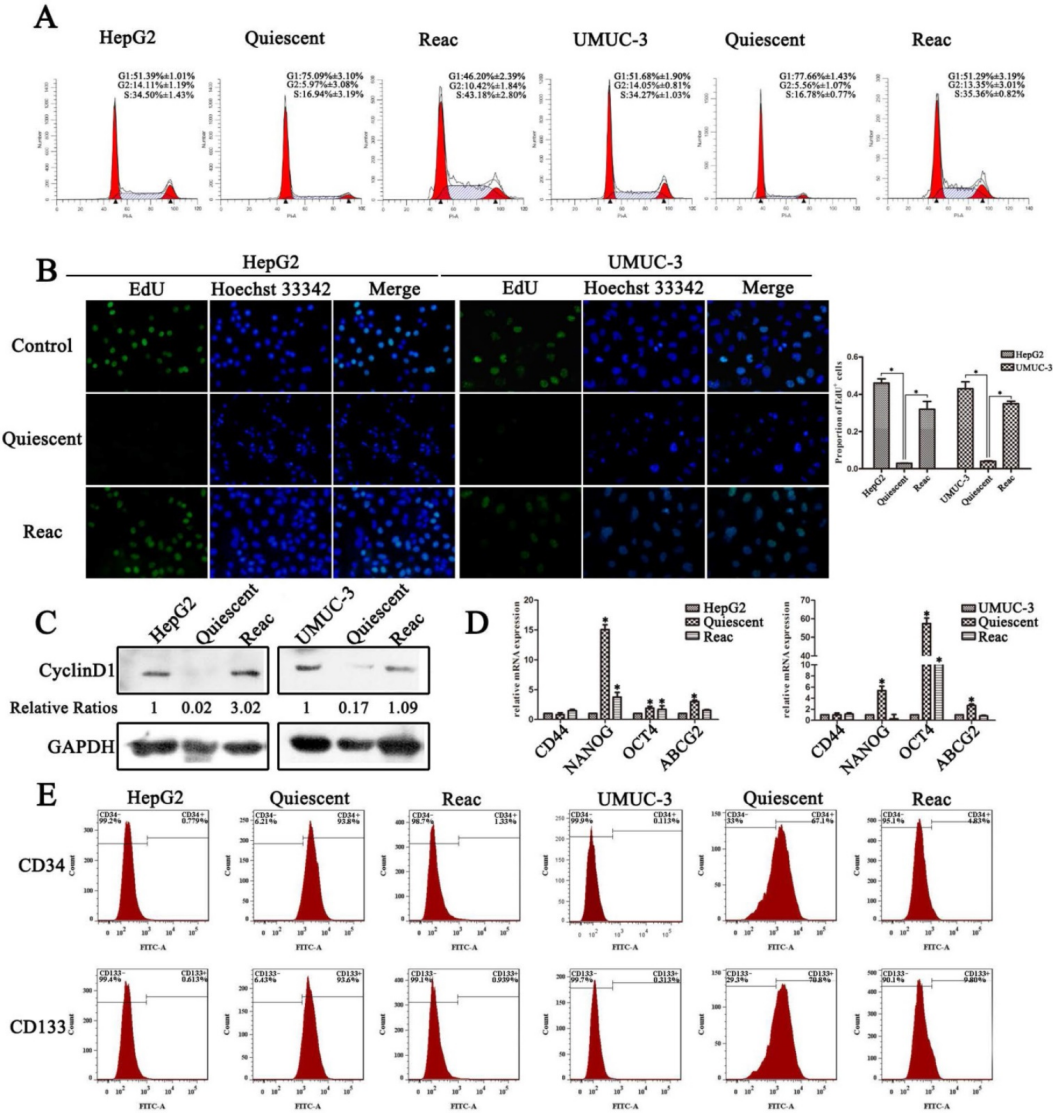
** PTX induces quiescent cancer cells with drug resistant capability and stem-like features.** Cells were treated with PTX for 7 days (Quiescent), then partially released into medium with half of the initial concentration of PTX and cultured for 3 days (Reac). (**A**) Cell cycle were analyzed by FACS. (**B**) Cell proliferation was detected by EdU incorporation assay. Data are shown as mean ± SD of three independent experiments, * *P*<0.05. (**C**)The expression of Cyclin D1 were detected by Western blot, GAPDH was used as loading control. (**D**) Stemness related genes expression were examined by real-time PCR. (**E**) CD34 and CD133 of cancer cells were detected by flow cytometry.

It has been reported by several groups that the multinucleated polyploid giant cancer cells (PGCC) contribute to produce of cancer stem-like cells and play a fundamental role in chemo-resistance in human cancer cells under replicative stress such as docetaxel 18-22. Our previous research also showed that cancer cells undergo mitotic slippage and generate PGCC after PTX treatment 23. In this research, we focused on the cells fate under long-term PTX stress. After PTX treatment for 7 days, G1/G0 instead of polyploidy or G2/M accumulation was observed (Figure 1A), DNA replication was dramatically decreased (Figure 1B), and the G1 specific Cyclin D1 was almost absent in the cells (Figure 1C). It appears that under continuous PTX tress cancer cells go into a non-proliferative quiescent state. Moreover, after partial PTX release (concentration of paclitaxel was reduced to half of the initial dose), these quiescent cells resumed proliferation (Figure 1A-C), indicating that these quiescent cells retain potential of reactivation.

These quiescent cells showed stem-like features, as confirmed by increased expression of the “stemness” gene NANOG, OCT4 and ABCG2 (Figure 1D), and higher percentage of CD34^+^/CD133^+^ population (Figure 1E). In quiescent HepG2 cell, NANOG is the most up-regulated gene, while the OCT4 gene expression increased most significantly in quiescent UMUC-3 cells. The expression of CD44 gene was not change significantly in both quiescent cells. After release, the reactivated cells lost stem-like features (Figure 1D and E). The loss of stemness may due to the mesenchymal to epithelial transition, which has been suggested to be required for reactivation of the stem-like circulating tumor cells 24, 25. However, although the reactivated cells lost stem-like features, these cells still manifested resistance to multiple anti-cancer drugs including PTX, vincristine and cisplatin (Figure S1).

### The reactivated cancer cells directly re-enter quiescence under higher PTX stress

**Figure 2:**
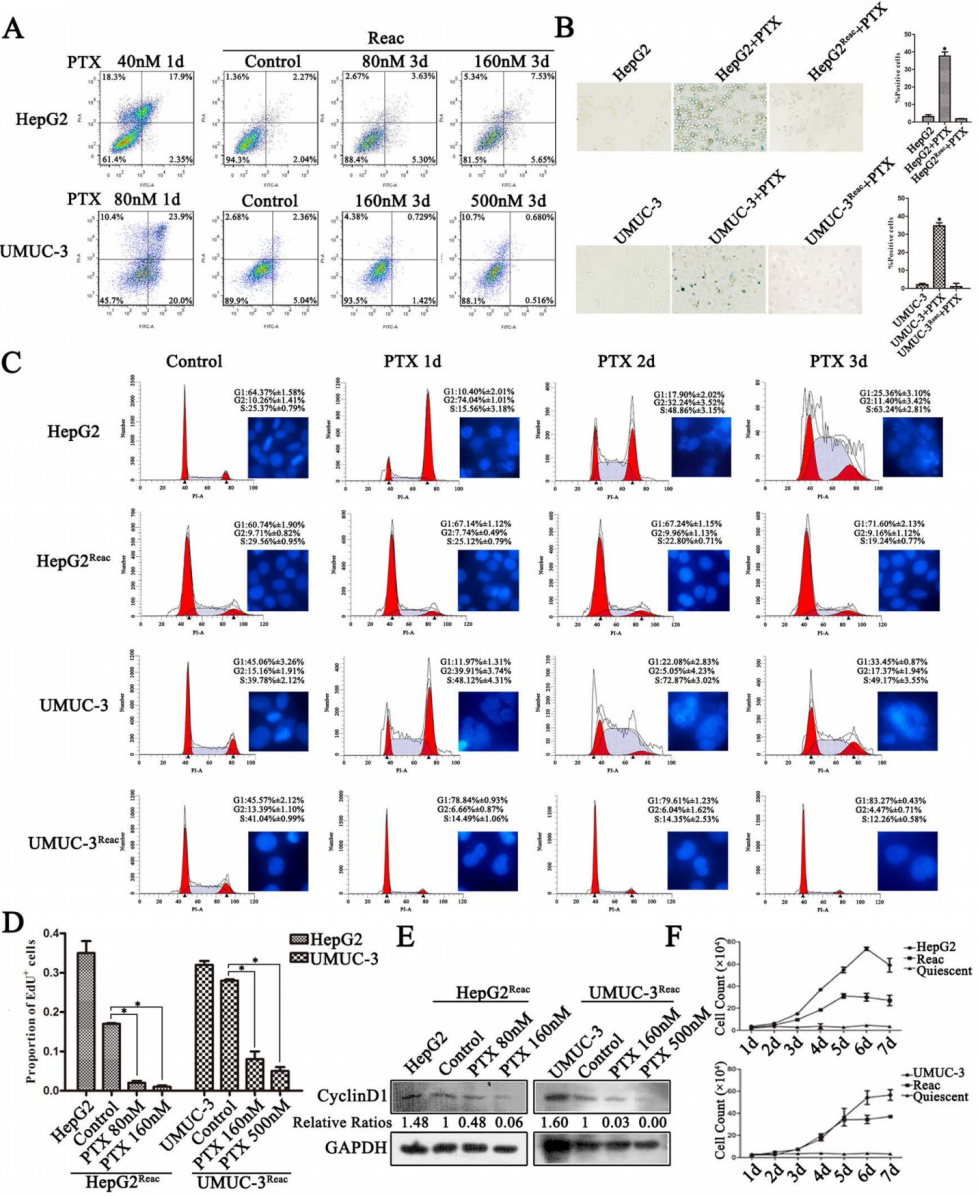
** The reactivated cells directly re-enter quiescence under higher dose of PTX without forming PGCC.** (**A**) The reactivated cells were treated with indicative concentration of PTX for 3 days. Cell apoptosis was examined by flow cytometry. Conventional cancer cells were treated with PTX for 1 day and used as positive control. (**B**) Cell senescence is detected by SA-β-Gal staining. Data are shown as mean ± SD of three independent experiments, * P<0.05. (**C**) Cells were treated with PTX (HepG2 40nM, HepG2^Reac^ 160nM, UMUC3 80nM, UMUC3^Reac^ 500nM) for 3 days, cell cycle profiles were analyzed by FACS and the cell nucleuses were visualized by Hoechst33342 staining. (**D**)The reactivated cells were treated with indicative concentration of PTX for 3 days, cell proliferation was detected by EdU incorporation assay. Data are shown as mean ± SD of three independent experiments, * *P*<0.05. (**E**) The expression of Cyclin D1 were detected by Western blot, GAPDH was used as loading control. (**F**) The cell growth curve during 7 days culture.

To characterize the chemo-resistance of these reactivated cells, we examined cell survival after 3 days of PTX treatment at higher doses than initial. Cell apoptosis was not observed at extremely high PTX concentration in the reactivated cells (Figure 2A). Under higher doses of PTX, on contrary to the control, the reactivated cells did not show G2/M arrest or polyploidy, but accumulated directly in G0/G1 (Figure 2C). Consistently, DNA replication was inhibited (Figure 2D, Figure S2) and Cyclin D1 protein was down-regulated (Figure 2E). Accordingly, the long-term growth of reactivated cells under higher PTX stress was significantly inhibited (Figure 2F). Moreover, the reactivated cells showed no sign of senescence under higher PTX stress (Figure 2B). This indicates that the reactivated cells readily re-enter quiescence to resist higher PTX stress.

**Figure 3:**
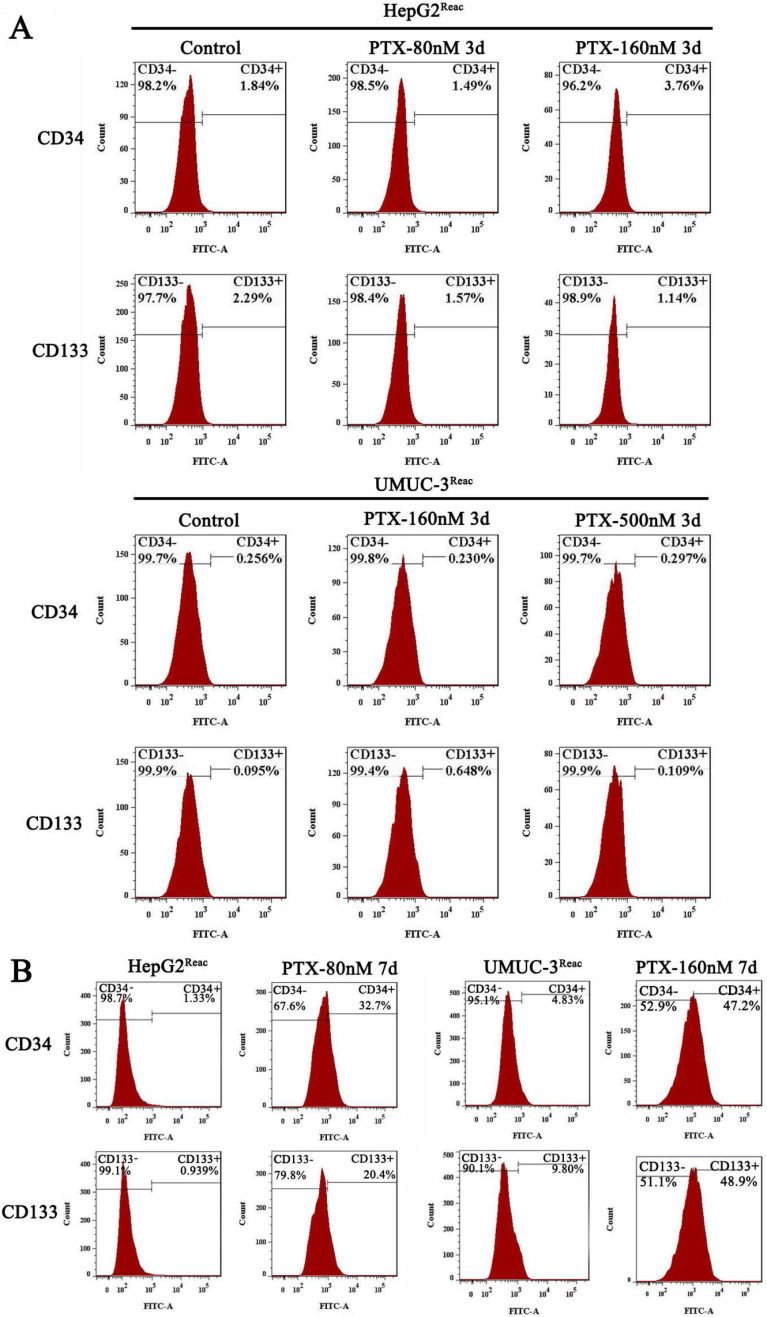
** Stem-like features of reactivated cells after 3 and 7 days at high doses of PTX.** (**A**)The reactived cells were treated with indicative concentration of PTX for 3 days, CD34 and CD133 expression were examined by FACS. (**B**) The reactived cells were treated with indicative concentration of PTX for 7 days, CD34 and CD133 expression were examined by FACS.

Interestingly, although the reactivated cells were in quiescence after 3 days of higher dose of PTX treatment, the populations of CD34^+^ and CD133^+^ cells did not change significantly at this time (Figure 3A). But after 7 days under higher PTX stress, the populations of CD34^+^ and CD133^+^ cells increased significantly (Figure 3B). This indicated the reactivated cancer cells are readily to enter quiescence, and subsequently obtain CSC-like characteristics during quiescence.

### Intracellular acidification leads cancer cells enter quiescence to resist PTX stress

**Figure 4:**
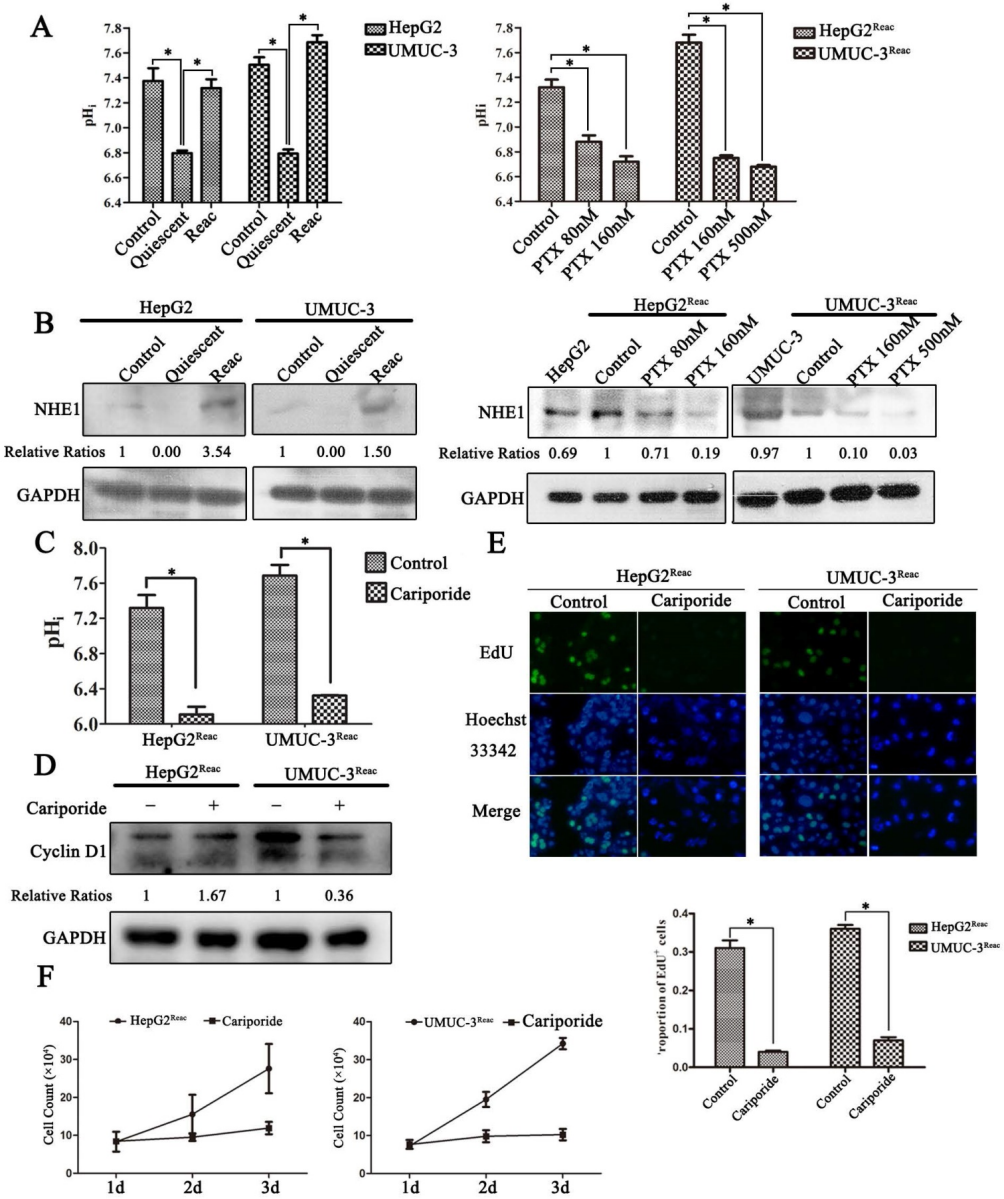
** The quiescent cancer cells are characterized with intracellular acidification.** (**A**) Cells were treated with PTX for 7 days then partially released to reactivation (left), the reactivated cells were treated with higher dose of PTX for 3 days (right), intracellular pH (pH_i_) was examined by BCECF-AM assays kit according to the manual protocol. Data are shown as mean ± SD of three independent experiments, * *P*<0.05, (**B**) NHE1 proteins in these cells were detected by Western blot. (**C-E**) The reactivated cells were treated with 100 µM Cariporide for 24h, (C) the intracellular pH was examined by BCECF-AM assays kit, data are shown as mean ± SD of three independent experiments, * *P*<0.05, (D)The expression of Cyclin D1 were detected by Western blot, GAPDH was used as loading control, (E) Cell proliferation was detected by EdU incorporation assay, data are shown as mean ± SD of three independent experiments, * *P*<0.05.(**F**) The cell growth curve during 3 days culture.

The pH homeostasis is critical for cell proliferation; we asked whether quiescence is associated with changes in pH_i_. As shown in Figure 4, the proliferative cancer cells had an alkaline pH_i_ (7.3-7.5), while in quiescent cancer cells the pH_i_ was acidic (6.7-6.8) (Figure 4A). Moreover, when the reactivated cells re-enter quiescence under higher dose of PTX pressure, the pH_i_ dropped gradually (Figure 4A). The reason for this intracellular acidification may be due to the downregulation of NHE-1 (Figure 4B), which is responsible for transporting hydrogen ions out of cells. More direct evidence showed that inhibition of NHE1 by cariporide produced intracellular acidification (Figure 4C), and could induce the reactivated cells re-enter quiescence (Figure 4D-F). Notably, cariporide treatment significantly decreased the protein level of Cyclin D1 in the reactivated UMUC-3 cells, but not in the HepG2 reactivated cells (Figure 4D). This may be due to the differences between cell types.

**Figure 5:**
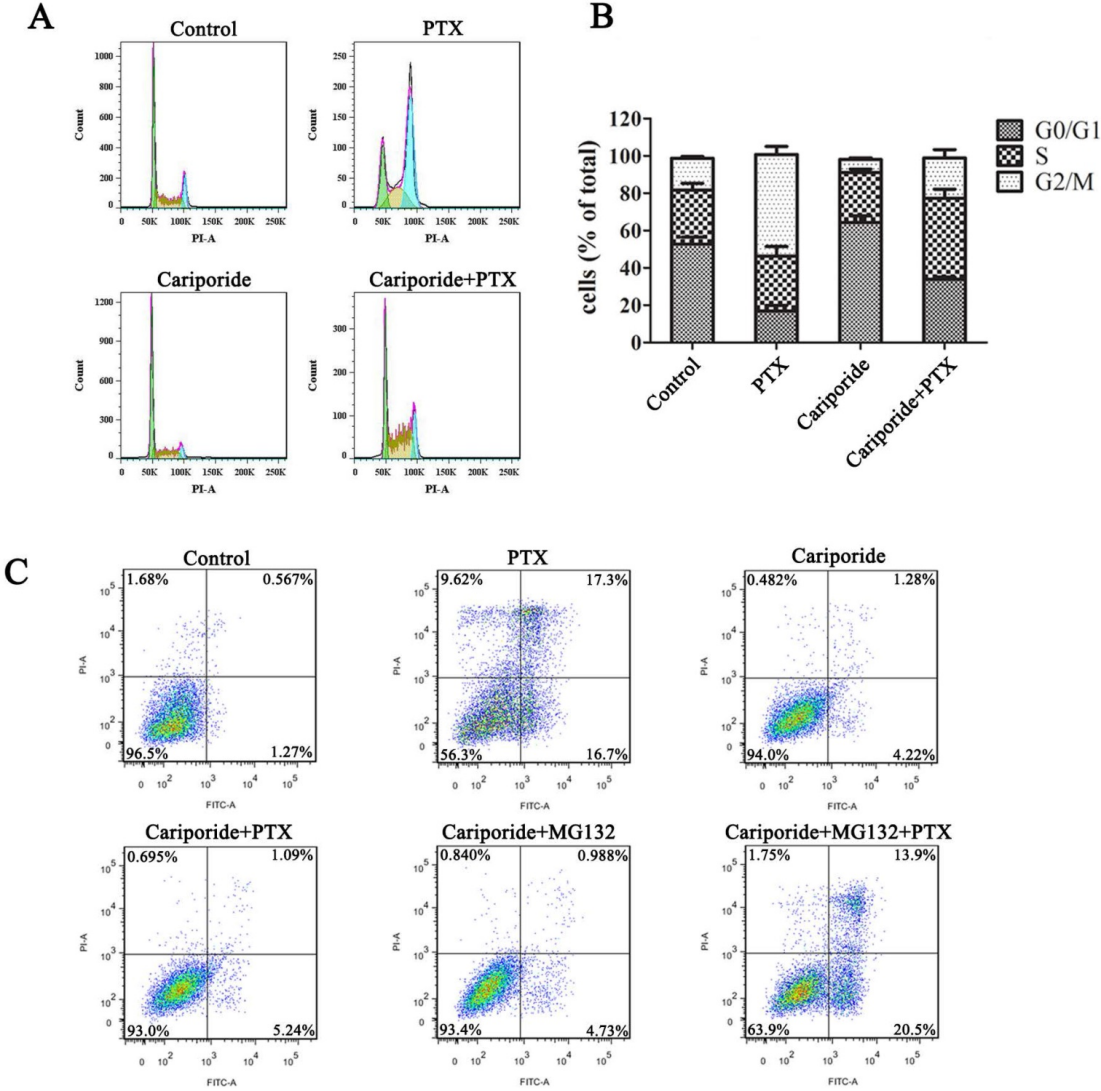
** Cariporide induces quiescence and PTX-resistance in UMUC-3 cells.** UMUC-3 cells were treated with Cariporide for 24h and then incubated with or without 80 nM PTX for 24 h. (**A**) Cell cycle was analyzed by flow cyctometry. (**B**) Histogram showed the proportion of cells in G0/G1, S and G2/M phases. (**C**) UMUC-3 cells were treated with or without Cariporide for 24h and then treated with 80 nM PTX combining with or without 1 µM MG132 for 24 h. Cell apoptosis was valued by flow cyctometry.

Next, we further clarified the relation of intracellular acidification, quiescence entry and PTX resistance in the parental cancer cells. Cariporide were added to UMUC-3 cells 24h before PTX treatment to induce intracellular acidification. As expected, cariporide pretreatment resulted in G0/G1 accumulation and abolished PTX-induced G2/M arrest in UMUC-3 cells (Figure 5A and B). Moreover, cariporide pretreatment resulted in resistance to PTX-induced apoptosis (Figure 5C). Thus, these results demonstrate that intracellular acidification could mediate cancer cells to enter quiescence to resist PTX killing.

### Intracellular acidification induces quiescence through promoting the protein ubiquitination process

**Figure 6:**
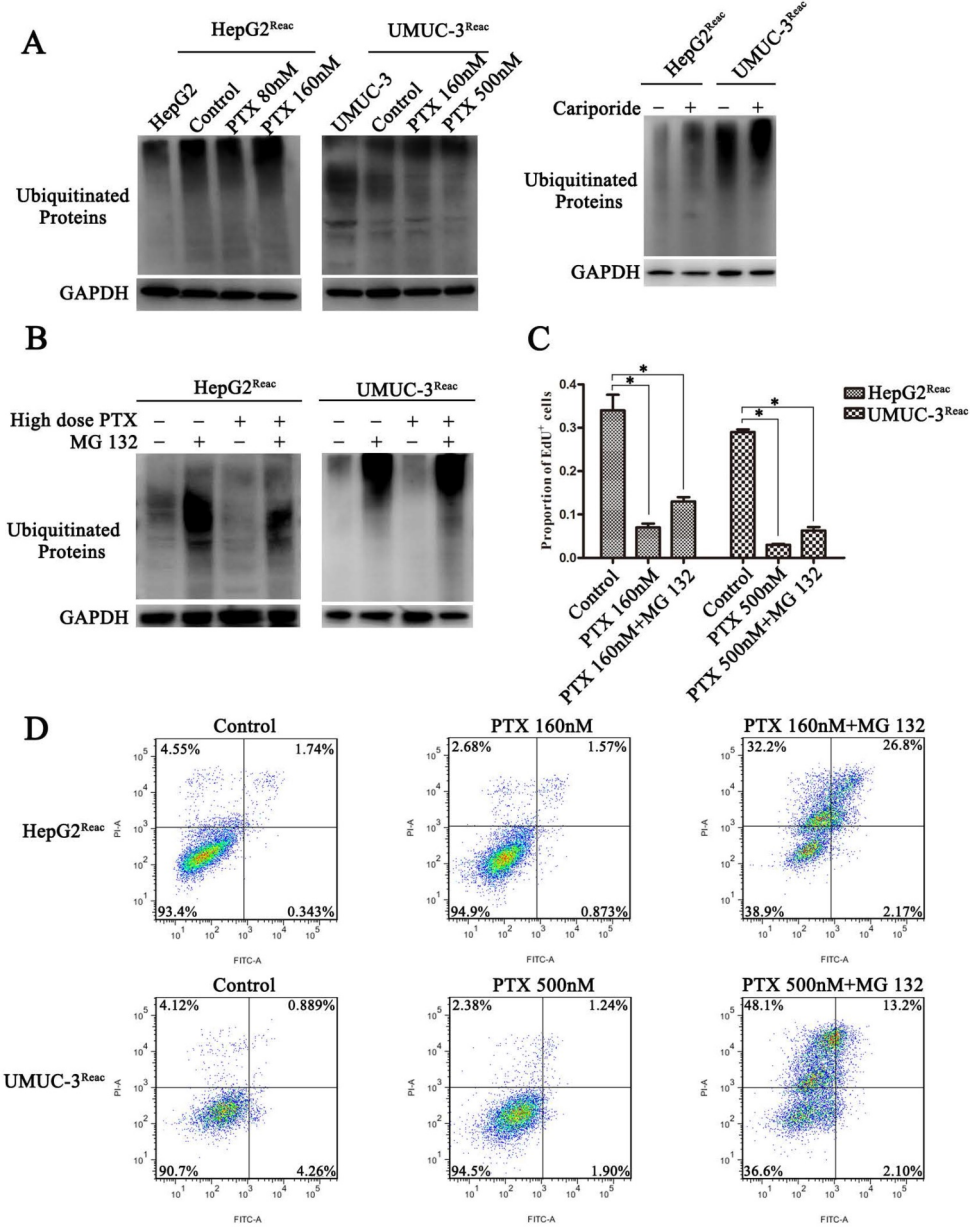
** Inhibiting proteasome activity impedes quiescence entry and increases drug sensitivity of the reactivated cells.** (**A**) The reactivated cells were treated with or without indicative concentration of PTX (Left) or 100 µM Cariporide (Right) for 24 h. Ubiquitination of cellular proteins was detected by Western blot using anti-Ub antibody. (**B-D**) The reactivated cells were treated with higher dose of PTX (HepG2^Reac^ 160 nM, UMUC3^Reac^ 500nM) combined with or without 1 µM MG132 for 24 h, (B) ubiquitination of proteins was detected by WB, (C) cell proliferation was detected by EdU incorporation, *p<0.05, and (D) cells apoptosis was examined by flow cytometry.

The ubiquitin-proteasome system (UPS) is a major way for cell degradation of key cell cycle proteins, and its activity is regulated by pH_i_ 26, 27. Therefore, UPS system may be engaged in acidic pH_i_-induced quiescence. As shown in Figure 6, the level of protein ubiquitination was increased in the acidic cells (Higher-dose-of-PTX- or cariporide-treated cells) (Figure 6A). The level of ubiquitinated proteins in the reactivated UMUC-3 was much higher than that in the reactivated HepG2, which may due to differences between cell lines (Figure 6A right panel). Moreover, the proteasome inhibitor MG132 further increased the level of ubiquitinated proteins (Figure 6B). This reveals a relation between intracellular acidification and protein ubiquitination process. Further, MG132 prevented the reactivated cells re-entering quiescence (Figure 6C and Figure S3), and restored cell sensitivity to PTX-induced apoptosis (Figure 6D). Similarly, in the parental UMUC-3 cells, MG132 reversed the cariporide-induced resistance to apoptosis (Figure 5C). These data indicate that intracellular acidification could enhance the UPS to induce cell enter quiescence. Our results also suggest that inhibition of the proteasome activity may be an efficient way to prevent cancer cells entering quiescence so as to enhance anticancer effect of PTX.

### MCM7 supports the reactivated proliferation of quiescent cancer cells

**Figure 7:**
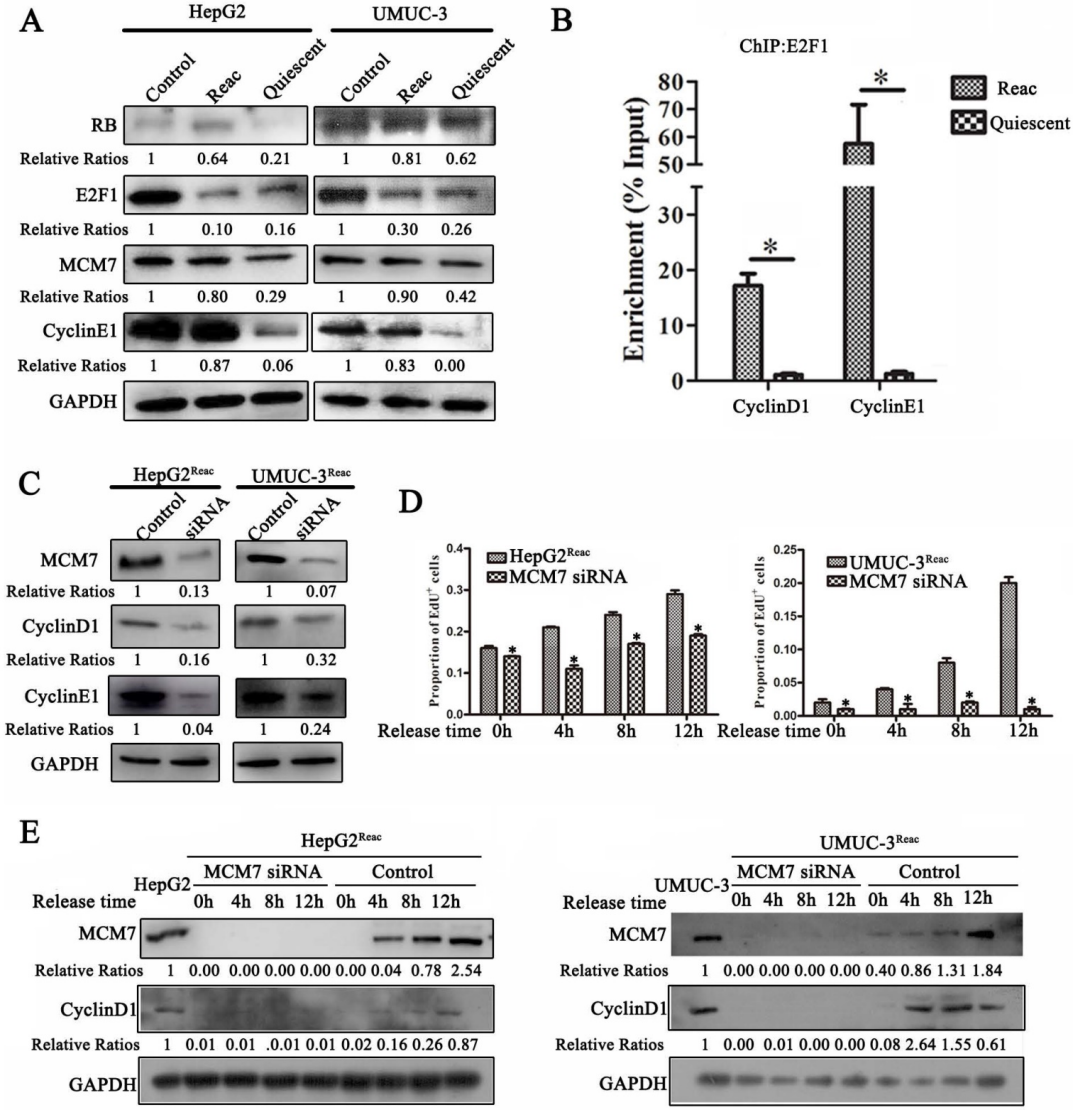
** MCM7 supports the reactivated proliferation of quiescent cancer cells.** (**A**) The reactivated cells were treated with or without higher dose of PTX (HepG2^Reac^ 160 nM, UMUC3^Reac^ 500 nM) for 3 days to induce quiescence, Western Blot were performed to examine the protein expression. (**B**) UMUC-3^Reac^ cells were treated with or without 500 nM PTX for 3 d, ChIP were done using an anti-E2F1 polyclonal rabbit antibody followed by PCR amplifying of cyclin D1 and E1 promoter regions. The ChIP results were obtained from three independent experiments, *P<0.05. (**C**) The reactivated cells were transfected with siRNA against MCM7 for 48 h. MCM7, Cyclin D1 and E1 proteins were detected by Western blot. (**D**) The reactivated cells were treated with PTX (HepG2^Reac^ 160 nM, UMUC3^Reac^ 500 nM) for 3 days to induce quiescence and then released into fresh medium, MCM7 siRNA was transfected 24h before the PTX-release, cells were collected at 0, 4, 8 and 12 h after the PTX-release, the cell proliferation was detected by EdU incorporation assay, data are shown as mean ± SD of three independent experiments, * *P*<0.05, and (**E**) MCM7 and CyclinD1 proteins levels were detected by WB. GAPDH was used as loading control.

Since the reactivated cells can proliferate under PTX pressure and tend to re-enter quiescence under higher stress, we speculate that these cells should have distinct proliferative mechanism. Therefore, we examined the G1/S transition proteins in the reactivated cells. Surprisingly, the protein level of E2F1 in the reactivated cells was much lower than that in the parental cells. It was identical to that in the quiescent cells (higher-dose-PTX-treated) (Figure 7A). However, ChIP assay showed that there were much more E2F1 binding to its target genes (cyclinD1 and cyclinE1) as compared to quiescent cells (Figure 7B). Moreover, in the reactivated cells, the protein level of E2F1 target gene cyclin E1 was increased to similar level as the parental cells (Figure 7A). This indicated that although E2F1 protein level was not recovered in the reactivated cells, its transcriptional activity is enhanced to start proliferation in the reactivated cells.

It has been reported that MCM7 participates in the regulation of RB·E2F1 complexes and helps G1-S transition 28-30, we speculate that MCM7 may support the reactivated proliferation. In support of this hypothesis, when quiescent cell reactivated, MCM7 protein was increased to identical level as it in the parental cells (Figure 7A). Moreover, MCM7 siRNA inhibited Cyclin D1 and Cyclin E1 expression in the reactivated cells (Figure 7C).

Next, we explore the effect of MCM7 depletion on the reactivation of quiescent cells. The reactivated cells were induced into quiescence via higher level of PTX treatment, and then cells were released into fresh medium for reactivation. MCM7 siRNA significantly inhibited EdU incorporation in the released cells (Figure 7D, Figure S4). Also, the expression of Cyclin D1 after release was completely inhibited by MCM7 siRNA (Figure 7E). These results indicate that depletion of MCM7 can suppresses the reactivation of quiescent cells and keep them at quiescent state.

## Discussion

The primary cause of tumor relapse after curative treatment is due to the existing of dormant cancer cells, which remain clinically indiscernible and asymptomatic 31-33. In this paper, after long-term PTX exposure, the residual survival cancer cells enter a non-proliferating state with extremely high drugs resistant capability. Moreover, after partial release, these cells resumed proliferating. This process can mimic, to some extent, the clinical appearance of cancer dormancy after curative chemotherapeutic treatment. We determine these non-proliferating cells are in quiescent or dormant state but not temporary G1 arrest by following reasons. Firstly, a long period of growth arrest was induced in this research. As known, long period of cell cycle arrest will lead to apoptosis or senescence 34. Secondly, these cells showed no sign of senescence and on contrary to cellular senescence, the non-proliferative state of these cells is reversible. Thirdly, the G1-S specific Cyclin D1 was completely degraded in these cells, indicating that these cells are not G1 arrest but be out of division cycle.

In this paper, long-term PTX-induced quiescent cells show stem-like feature. Moreover, the change of stem-like markers is different between two cell lines. NANOG is the most up-regulated gene in quiescent HepG2 cell, while the OCT4 gene expression increase most significantly in quiescent UMUC-3 cells. This is consistent with previous reports showing that cancer cells enter quiescent state with stem-like phenotype under environmental stress such as 5-FU 35 and chronic hypoxia 36. Also, the clinical separated DTCs have been identified to have both quiescent and stem-like features 37, 38. Thus, it appears that tumor cell quiescence is closely associated with stemness. In addition, in this study, after partial release, cells are reactivated to proliferate but lost stem-like feature. Further, when re-challenged with second PTX stress, the reactivated cells re-enter quiescence and subsequently obtain stem-like feature. This suggests that quiescence is an adaptive state to resist PTX stress, in which cancer cells subsequently undergo further malignant transformation.

The molecular signature of quiescent cancer cells is currently poorly understood. In this research, we find quiescent cancer cells are characterized by apparent intracellular acidification. Our results demonstrate a supporting role of acidic pH_i_ on quiescence entry. This is consistent with previous reports that acidic pH_i_ can inhibit the Raf/MEK/ERK pathway 39, which is a characteristic of dormant cancer cells 40. Our results also reveal that acidic pH_i_ could enhance whole cell protein ubiquitination. It has been reported that PTX plus MG132 treatment is more effective than PTX alone in inhibiting the aggressive phenotype of breast cancer cells 41. Also, the combination of paclitaxel and proteasome inhibitor 1(PS1) may be a new cancer treatment strategy 42. These results indicate proteasome inhibition potentiates the anti-cancer efficacy of other chemotherapeutic drugs. Recently, an interesting report in quiescent yeast shows that acidic pH_i_ induces widespread macromolecular proteins assembly and leads to a solid-like cytoplasm with reduced mobility 43. Therefore, the acidic pH_i_, as a widespread intracellular environment, could impact many aspects of intracellular activities such as growth signal transduction, protein stability and ubiquitination, which merits further exploration 44.

In this research, we find that E2F1 protein level is not recovered in the reactivated cells. Its content is equal between the reactivated and quiescent cells. However, there are much more E2F1 binding to its target genes (cyclinD1 and cyclinE1) in the reactivated cells, suggesting that its transcriptional activity is enhanced in the reactivated cells. On the other hand, we find that MCM7 protein level is recovered in the reactivated cells. Moreover, MCM7 depletion could inhibit G1/S genes transcription and prevent the reactivation of quiescent cell. Thus, it seems that MCM7 function in the G1-S transition and promote the reactivated proliferation. This is consistent with previous studies showing that MCM7 has a regulatory effect on the RB-E2F complex, thereby promoting G1-S transition 28, 29. It has been reported that MCM7 associate with RB and is required for RB's efficient inhibitory effect on DNA replication 28. Dissociation of RB-MCM7 complex is catalyzed by the Cyclin D1/CDK4 to promote G1-S transition 29. Recent study show that decreasing MCM7 can inhibit the proliferation of Rb-deficient cancer cells 30. Considering the excessive MCMs binding to chromatin and the involvement of MCM7 in the RB·E2F1 complexes 28, 29, we suppose that the excessive chromatin-binding MCM7 may be an alternative way to initiate proliferation when cells are under stress.

## Supplementary Material

Supplementary figures and tables.
