# Supplementary material for: Cancer Cell enters reversible quiescence through Intracellular Acidification to resist Paclitaxel Cytotoxicity
Source: Int J Med Sci. 2020 Jun 29;17(11):1652–64. doi: 10.7150/ijms.46034 (PMC7359388; doi:10.7150/ijms.46034)
Supplement: Supplementary file 1 — Supplementary figures and tables. [file ijmsv17p1652s1.pdf]

Table 1 Primers for real-time PCR

Gene Name	Forward(5'-3')	Reverse(5'-3')
CD44	CTGCCGCTTTGCAGGTGTA	CATTGTGGGCAAGGTGCTATT
NANOG	AAGGTCCCGGTCAAGAAACAG	CTTCTGCGTCACACCATTGC
OCT4	CTGGGTTGATCCTCGGACCT	CCATCGGAGTTGCTCTCCA
ABCG2	CAGGTGGAGGCAAATCTTCGT	ACCCTGTTAATCCGTTCGTTTT
ABCC1	CTCTATCTCTCCCGACATGACC	AGCAGACGATCCACAGCAAAA
ABCB1	TTGCTGCTTACATTCAGGTTTCA	AGCCTATCTCCTGTCGCATTA
TOP2B	AGCCATTGACGCAGTTCATGT	CCTGGCACAAAGGTAACCTCC
GSTK1	TCTGGAAAAGATCGCAACGC	GCCCAAAGGCTCCGTATCTG
β-actin	ACAGAGCCTCGCCTTTGC	GCGGCGATATCATCATCC

Type	PTX (nM)	Cisplatin (μg/ml)	Vincristine (nM)
HepG2	21.56 ±1.17	2.42 ±1.15	60.64 ±1.46
Quiescent	3185 ±1.94	2.42 ±1.03	1848 ±1.42
Reac	839.7 ±1.09	1.86 ±1.01	794.8 ±1.37
UMUC-3	290.1 ±1.06	4.48 ±1.32	0.89 ±1.22
Quiescent	> 5000	10.67 ±1.13	16.83 ±1.38
Reac	4769 ±2.10	14.23 ±1.17	45.36 ±1.92

Figure S1. long-term PTX treatment induces multiple anti-cancer drugs HepG2 and UMUC-3 Cells were treated with indicative anti-cancer drugs (PTX, vincristine and cisplatin) for 2 days; cell viability was valued by MTS assay. The data are shown as IC_{50} (mean ±SD of three independent experiments).

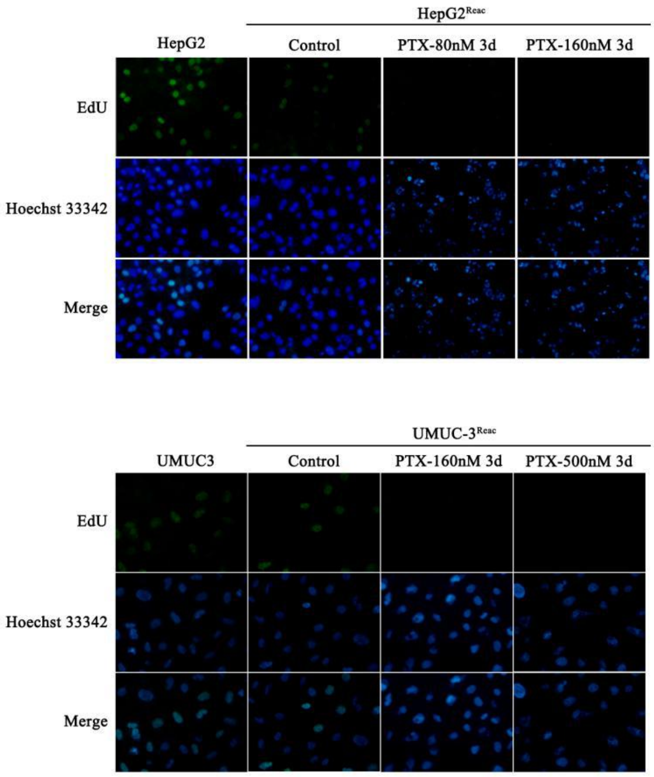

Figure S2. Higher doses of PTX readily induces quiescence of the reactivated cells. The reactivated cells were treated with indicative concentration of PTX for 3 days, immunofluorescence staining was performed to detect EdU (green) and nucleuses were visualized by Hoechst33342 staining (blue).

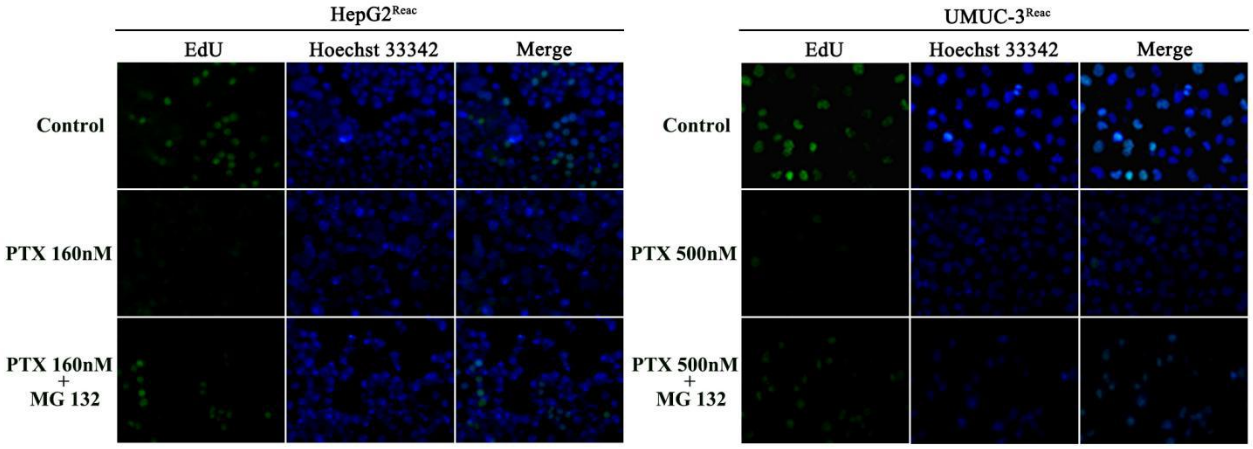

Figure S3. MG132 inhibits reactivated cell entering quiescence. The reactivated cells were treated with high dose of PTX (HepG2^{Reac} 160nM, UMUC3^{Reac} 500nM) combined with or without 1μM MG132 for 24h, immunofluorescence staining was performed to detect EdU (green) and nucleuses were visualized by Hoechst33342 staining (blue).

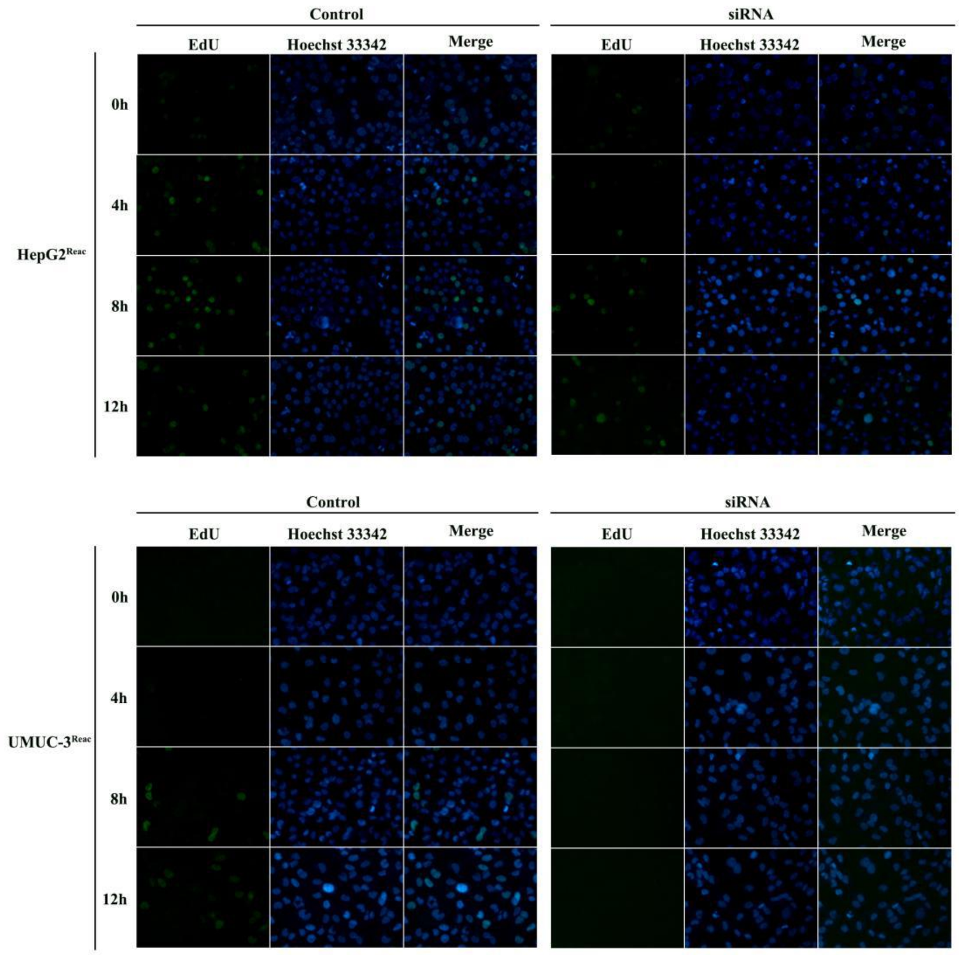

Figure S4. MCM7 siRNA inhibits the reactivation of quiescent reactived cells. The reactivated cells were treated with PTX (160nM for HepG2[Reac], 500nM for UMUC-3[Reac]) for 3 days to induce quiescence and then released into fresh medium, MCM7 siRNA was transfected 24h before the PTX-release, cells were collected at 0h, 4h, 8h and 12h after the PTX-release, immunofluorescence staining was performed to detect EdU (green) and nucleuses were visualized by Hoechst33342 staining (blue).
